# Do young black men who have sex with men in the deep south prefer traditional over alternative STI testing?

**DOI:** 10.1371/journal.pone.0209666

**Published:** 2018-12-27

**Authors:** Ellen F. Eaton, Erika L. Austin, Catherine K. Dodson, Jose P. Heudebert, D’Netria Jackson, Christina A. Muzny

**Affiliations:** 1 University of Alabama at Birmingham, Department of Medicine, Division of Infectious Diseases, Birmingham, AL, United States of America; 2 University of Alabama at Birmingham, School of Public Health, Department of Biostatistics, Birmingham, AL, United States of America; 3 University of Alabama at Birmingham, School of Medicine, Birmingham, AL, United States of America; Hofstra University, UNITED STATES

## Abstract

**Background:**

High sexually transmitted infection (STI) rates in the South, especially among young black men who have sex with men (YB MSM), make STI testing imperative for public health.

**Purpose:**

To identify STI testing preferences in this population to improve testing delivery and utilization.

**Methods:**

YB MSM ages 16–35 in Birmingham, Alabama participated in focus groups (FG). A trained qualitative researcher coded transcripts after each FG and added questions to explore emerging themes.

**Results:**

Between September 2017 and January 2018, 36 YB MSM participated in 5 focus groups. Median age was 25.5 (Interquartile range 22–30). Participants preferred STI testing at doctors’ offices conducted by physicians but they also preferred having options related to testing locations, frequency, and timing to address diverse needs. Participants did not prefer testing by non-physician staff or home self-testing.

**Conclusion:**

A variety of options, including varied locations, personnel, and methods (self-collected and provider collected) are needed to make patient-preferred STI testing a reality among YB MSM in the Deep South. Further, the desire to be tested by a trusted physician highlights the need for access to primary care providers. Results suggest that newer home-based tests and self-collected tests are less preferable to YB MSM in the South, which deserves further study as these tests are rapidly integrated into clinical care.

## Introduction

Sexually transmitted infections (STIs) are on the rise, especially in men who have sex with men (MSM), and contribute to the 45,000 new HIV infections annually by increasing HIV transmission risk [1, 2]. Alabama ranks 2^nd^ and 3^rd^ nationally in gonorrhea and chlamydia incidence, respectively [2]. Nationwide, the annual incidence of chlamydia, gonorrhea, and syphilis in MSM are currently 7.1, 6.4, and 3.4 per 100 person years, respectively [3]. Through multiple mechanisms, STIs increase the likelihood that men will acquire HIV or transmit HIV if already infected [4]. Young persons ages 16 to 35 are at greatest risk for HIV, representing over 50% of new infections [5]. Furthermore, 1 in 2 black MSM will acquire HIV during their lifetime if current trends continue [5]. Reducing STI transmission among YB MSM is critical to halting the HIV epidemic in the Deep South. The first step in combatting STIs is early diagnosis to prevent transmission. Yet, research on STI testing preferences is limited in YB MSM including preferences for testing location, staffing, and hours of operation for STI testing sites [6, 7]. There is a critical need for research on the most accessible STI testing services from the perspective of high-risk individuals, including hard-to-reach YB MSM who have unique barriers to STI testing. Some of these barriers include poverty, abstinence-only sex education, and increased STI-related stigma in the Deep South compared to other regions. These factors have been shown to negatively affect STI testing and treatment [8–10]. Specifically, young blacks in the Southeast experience more HIV-related stigma compared to those in the Northeast, and black MSM may experience more anti-gay stigma than white MSM [11, 12]. Determining and implementing patient-centered STI testing options may circumvent some of the complex societal barriers experienced by this vulnerable population.

Eliciting STI testing preferences from the perspective of those at greatest risk is essential to identifying the most preferable ways to incorporate novel test delivery methods (ie. Home-based, self-collected) into routine care. Furthermore, this work is needed in the Deep South, which is at the epicenter of STI and HIV epidemics nationally. With self-collection, STI testing can reliably and feasibly be provided in non-clinical settings including community-based organizations [13]. Highly sensitive and specific nucleic acid amplification tests (NAATs), available for the detection of chlamydia and gonorrhea, can be performed on self-collected specimens (i.e. urine, rectum, oro-pharynx) obtained in a variety of locations, including the home [13–15]. Research in some settings has shown that self-collection and privacy are priorities for patients but this work has not been conducted in the Southern U.S [16, 17]. Although syphilis testing is not currently feasible by patient self-collection, a point-of-care testing option (ie. fingerstick for serological testing) can be conducted in non-clinical settings [18]. Self-collection may allow young MSM who are unwilling and/or unable to access traditional clinical settings (due to stigma, transportation, and work-related barriers) to obtain appropriate STI care, reducing the number of undiagnosed STI infections and transmissions [6].

Identifying patient preferences related to STI testing in the Deep South may shed light on ways to increase testing enabling more rapid STI diagnosis and treatment. One patient-centered STI testing initiative in the United Kingdom (U.K.), which includes both a clinic and a home-based testing outreach, has increased STI screening capability two-fold [19]. All aspects of the clinic including the location, staff, technology, and communication methods are designed to fit the needs of urban clients at risk for STIs. As a result, clients report that staff attitudes, attire, and professionalism contribute to a relaxed, non-judgmental approach to STI care making them more likely to return and recommend this clinic to others. This clinic has relied on specimen collection by clients and less skilled staff in order to free up clinicians’ time for treatment and counseling. This non-traditional approach, whereby support staff perform many testing duties, has increased efficiency and testing capability. As a result, this clinic screened almost 8,500 individuals monthly and 11.5% of individuals tested positive for at least one STI. A total of 85 incident HIV diagnoses were made via the home-based testing initiative in 2 years [19]. The success of this initiative highlights that patient-centered STI testing can increase test utilization and STI diagnoses and positively impact the STI/HIV epidemic in MSM in the U.S. Before implementing a patient-centered STI testing initiative in the Deep South, it is imperative that we address research gaps on patient values related to testing location, procedures, and personnel according to the preferences of those at greatest risk in this region.

The objective of this study was to identify the STI testing preferences of young Black, MSM in the Deep South to inform a testing initiative in this vulnerable population. The rationale is that identifying the most acceptable STI testing options will allow development and implementation of patient-centered STI testing services, which will be more likely to be utilized among YB MSM.

## Materials and methods

We recruited YB MSM ages 16–35 in Birmingham, Alabama to participate in focus groups (FG). Inclusion criteria included self-identification as African-American, male, ages 16–35, and a history of sex with men; all participants agreed to study procedures, including permission to audiotape the focus group. Young black male participants were recruited through flyers posted in and around the Birmingham metropolitan area. Flyers were displayed at local emergency departments, the Jefferson County Health Department STI Clinic, and clinics serving the University of Alabama at Birmingham (UAB) community (Student Health Clinic, the Lesbian, Gay, Bisexual, and Transgender (LGBT) Psychiatry Clinic, the Adolescent Health Clinic, the Graduate Student Counseling Clinic, student dining halls, and dormitories), free medical clinics, Birmingham AIDS Outreach, AIDS Alabama, bus stops, and other sites (bars and clubs, coffee shops). An advertisement was also placed in the UAB e-Reporter, an electronic mailer that goes to UAB employees monthly. A young, male study staff member recruited at gay bars, clubs, and local community events including the National Association for the Advancement of Colored People (NAACP) block party, the Magic City AIDS Walk, and Birmingham AIDS Outreach Bingo nights. He also communicated with UAB student organizations to increase study awareness. Eligible participants were encouraged to share study information with peers, allowing “word of mouth” recruitment. Research staff at the UAB HIV/AIDS clinic also assisted with “word of mouth” recruitment. Eligible persons were required to provide written informed consent prior to any research activities. A $30 cash incentive was given for participation. All study activities and forms were approved by the University of Alabama at Birmingham Institutional Review Board (protocol # 170530004).

Prior to participation in a FG discussion, all participants completed a standardized survey on their socio-demographics (e.g., age, race/ethnicity), recent sexual history, and STI history, including HIV. Using a standardized script, an experienced moderator gave FG participants an introduction to the types of STI testing services that are currently available (see S1 Fig). Participants were then asked a series of questions related to STI testing with an emphasis on location, personnel, and available STI tests (e.g., urine, oral/rectal/urethral swabs, and blood tests). Toward the end of each FG, participants were asked to describe their ideal testing experience, including what other services would be offered concurrent with testing. Probes were used to encourage responses related to emerging themes such as perceived stigma at testing sites. All FG were facilitated by the same moderator skilled in qualitative research methods. Each FG was limited to 60 minutes. Each FG was audio-recorded and transcribed by a third party professional. Transcripts were reviewed by the research team in a debriefing meeting following each FG, and additional questions were added to explore emerging themes. Transcripts were thematically coded by a trained qualitative researcher using a systematic process (ELA). Based on the debriefing meeting discussions, a list of initial codes was developed to sort the data into broad categories roughly matching the focus group script (e.g., where patients prefer to be tested). A second level of thematic codes (e.g., experience of stigma) was then generated and applied across all transcripts to illustrate the participants’ overarching concerns and preferences regarding STI testing.

## Results

In total, between September 2017 and January 2018, 36 YB MSM participated in 5 FG (3 to 9 participants each). The median age and interquartile range (IQR) were 25.5 and 22 to 30, respectively (Table 1). A summary of the ages and number of participants in each group is included in the supplemental file (S2 Fig). Overall, five participants (14%) reported 5 to 10 sexual partners in the last 6 months. Many (n = 17, 47%) had repeatedly engaged in STI testing (≥5 times) and reported < 5 previous STI diagnoses (n = 35). Sixteen were HIV+. Below is a summary of the major themes, which emerged when participants were asked to share their preferences related to 1) locations where testing takes place, 2) the types of personnel by whom testing is conducted, and 3) views on methods of testing.

**Table 1:** Summary of YB MSM participating in an STI testing study.

**Characteristic**	**n**	**%**
Age		
17 and under	1	-2.8
18 to 24	15	-41.7
25 to 30	12	-33.3
Over 30	8	-22.2
Median age (IQR)	25.5	(22–30)
Sexual Orientation		
Homosexual	26	-72.2
Heterosexual	1	-2.8
Bisexual	9	-25
Number of sexual partners (past 6 months)		
Less than 5	30	-83.3
5 or more	6	-16.7
Number of prior STI tests		
Less than 5	19	-52.8
5 to 9	8	-22.2
10 or more	9	-25
Number of prior STI diagnoses		
Less than 5	35	-97.2
5 or more	1	-2.8
HIV positive		
Recruitment site		
Clinical site	10	-27.8
Referral	12	-33.3
Community site	11	-30.6
College campus	2	-5.5
Monthly university employee newsletter	1	-2.8

Clinical site refers to participants who were recruited by staff at outpatient clinics.

Referral refers to participants who were recruited via flyer or by word of mouth.

Community site refers to participants referred by staff or clients at sites affiliated with AIDS Alabama and Birmingham AIDS Outreach.

### Multiple options for STI testing

Across all FGs, the importance of multiple options for STI testing was consistently reiterated. Participants highlighted the numerous ways in which members of their community differ with regard to their reasons for and attitudes about testing and suggested that comprehensive testing programs would need to address these diverse needs. For example, some individuals are regular testers (undergoing STI testing every three months) while others seek out testing immediately prior to or following a sexual encounter or when notified of a possible exposure through contact tracing. Participants noted that while some individuals rely on a single, consistent place for testing, others may be encouraged to test when an unexpected opportunity for testing is presented (at an event, for example). One participant stated:

*“I would definitely agree with both [testing locations] because you’ll always have the audience who it’s*, *well*, *I need to get tested*, *so I know that this place is for that*. *Let me go to this place*. *Then you also have individuals that it would definitely be a benefit of having it available in unconventional places*.”

An individual’s age and access to financial resources (including health insurance) were also frequently mentioned by participants as critical determinants of STI testing preferences and practices in this community.

### Private yet visible and normal

Views on the appropriate degree of visibility around testing differed considerably. While all participants expressed a general desire for privacy in STI testing, there was a distinct subset of participants who saw value in greater visibility around STI testing. Many expressing this view were leaders in their community due to their association with community centers or their role as parents in families of choice [20,21]. One participant went so far as to propose that stickers be given out at STI testing sites proclaiming “I just got tested,” similar to the stickers given after voting in many locales. The goal of such visibility appeared to be the normalization of testing, which many viewed as critical given the pervasive stigma around same-sex relationships (or even sexual relationships outside of heterosexual marriage) in the African American community.

### The importance of legitimacy

In discussing different testing locations, personnel, and methods, the importance of legitimacy emerged as a central concern. Although participants in this study were relatively knowledgeable about STI testing, there remained considerable confusion and misinformation about the sensitivity of self-collected STI tests and the indications for different STI testing methods. Perhaps recognizing this limitation, participants expressed a clear preference for more professional, clinical settings and procedures. A related concern was the availability of treatment in addition to testing; many participants expressed the view that while anyone can be trained to conduct tests and deliver results, only qualified professionals can prescribe or administer treatment.

### Preferred testing locations

#### Private doctors’ offices

Participants were in agreement that private doctor’s offices were the ideal location for STI testing, though they noted that this option was not available to individuals without insurance or the resources to cover co-pays or out-of-pocket expenses. Privacy was noted as the primary benefit of seeing a private doctor, and the sense of privacy was further enhanced with primary care providers who could be offering treatment for any condition.

*“…The private doctor, it’s like, you just going for your normal–you could be there for a cold, or whatever*.*”*

While privacy seemed most critical before and during STI testing, confidentiality when receiving results was also a central concern. Many participants noted that doctors and nurses are required to maintain confidentiality, with some participants even mentioning professional ethics and HIPAA regulations surrounding confidentiality.

*“Well, I like the medical because of the whole confidentiality thing. It makes me feel better knowing that I’m talking to a medical professional*.*”*

*“I do agree though, definitely, medical people ‘cause they’re bound by law to keep everything confidential. You have somebody that’s trained to give the test*, *but…*

*But by law, if they give ‘em the test*, *then they have to follow the HIPAA law also, don’t they?”*

Private doctors’ offices were also perceived to be one of the most comfortable options, which was considered important given that many individuals are scared by testing procedures or the possibility of receiving positive test results. Many participants spoke of having long-standing relationships with doctors, with some younger participants even mentioning having been tested by their pediatricians in the past. Ongoing relationships, along with respectful treatment and culturally-competent care, help to establish a sense of trust that participants value greatly.

*“If it’s your primary doctor, you’ve been going there for quite some time… You’ll be more comfortable in that setting and you’re already comfortable with them, versus going to a whole new place, getting tested by someone you don’t know and don’t trust*.*”*

#### General health clinics

General health clinics (often referred to as “Doc-in-a-Box” clinics) were also strongly endorsed as sites for STI testing. Like private doctors’ offices, these clinics had the benefit of offering care for a wide range of concerns, so testing can be sought privately. For individuals without access to a long-term relationship with a private doctor, these clinics were viewed as an excellent alternative.

“*Back to the envisioning of a perfect place…if it’s like a Doc-in-a-Box that also does HIV testing or STI testing*, *just because you know I’m there doesn’t mean I’m going for a test*. *I may be going because I have a cold or I have a headache or I need medicine or something like that*. *Passing by*, *I see your car there…Oh*, *he just sick today*.*”*

#### The local health department

Views on visiting the local Health Department for STI testing were more varied, with strong opinions on both the benefits and drawbacks of testing at this location. Others noted that as a government agency, care at the Health Department often feels impersonal and uninformed.

*“It’s the way they treat you, I guess. It’s just like you’re a number, in a sense, and I think they lack a sense of compassion or anything. It’s just like OK, we have to do this test and we’re just doing our job*.*”*

Participants all agreed that while the environment was less than desirable, the low or no cost of testing and the availability of immediate treatment balanced out concerns about location. Likewise, while many expressed frustration with the local Health Department’s current scheduling process (which involve calling early in the morning for a same-day appointment) and limited hours, most felt that STI testing was readily accessible through the local Health Department for those who understand the process of accessing care.

The most prevalent concern with STI testing at the local Health Department related to multiple forms of stigma. There was strong agreement that testing at the Health Department offered no privacy, as anyone seen there would automatically be assumed to be seeking STI testing and unable to afford doing so in a more private setting. For YB MSM, there are additional forms of stigma associated with being seen at the local Health Department:

*“Right, but primarily, if you’re at the Health Department… Everyone basically knows that that’s what you’re there for. I think looking at it systemically and looking at the social side of it, it’s like, Okay. Well, if I’m at the Health Department, that means that it carries the perception that I don’t have money to see a private doctor, which means that I’m poor and now I’m being tested for this health condition and I’m poor. That’s the talk that’s in the community when we talk about the Health Department. Now it’s two stigmas layered. Right? On top of each other. It’s the broke, black gay man. Yeah, the broke, black gay man. That’s who goes there. Who may have a disease*.*”*

#### Home testing

Views on STI testing at home were predominantly negative and reflected a different assessment of benefits and drawbacks than the other testing options. The potential convenience of testing at home was highlighted by one participant, though it was qualified with concerns about the accuracy of such testing:

*“I know the convenience of that would be for me not having to sign in, sit in the waiting room and wait. If I could go ahead and do this at home, especially with proper instructions, I’d go ahead, get the specimen, and drop it off. It’s definitely convenient*.*”*

Most participants reported feeling unsure if at-home testing would be accurate enough. Many mentioned it could be used as a preliminary test before going for more formal testing by a health care provider, similar to a home pregnancy test. One participant suggested that at-home testing might be used by individuals who are scared to get tested as a “gateway,” or a chance to test privately and process the results before having them confirmed.

*“I did it because I had always planned on going to get tested somewhere else, but I gave me, when I did it at home and I got the results, it kinds eased by mind going into it…People can be at home, and they can kinda do it, and they can kinda prepare themselves, because once you know, you know*.*”*

Still others worried that testing at home might actually have the unintended consequence of encouraging people to forgo treatment. Several participants described scenarios in which people may learn of their HIV status at home but fail to change their sexual risk behavior as a result. Others worried that individuals choosing to test at home might be emotionally unprepared to deal with a positive test result without the support of a trained testing counselor.

*“I’m indifferent about at-home testing and reason being is because, well, my worry is if the test is reactive*, *will you follow up and will you be strong enough to follow up?”*

*“You don’t have any social support at home, so you’re facing this preliminary diagnosis on your own*. *What type of headspace does that out you in?”*

Several other testing locations were discussed by study participants, including places where testing is offered and places where testing might be effective for increasing testing in their community. Views on testing at clubs were split, with some saying that patrons would be resentful of having their fun night ruined by being forced to think about testing. Others said it would be a great opportunity for opinion leaders to get visibly tested and lead by example. Likewise, attitudes about testing at churches were mixed, though everyone agreed that only certain African American churches would be open to this. Some believed that the church should engage congregants for STI testing, others thought that testing at churches would not be kept confidential. One recommendation that emerged across all discussions was the idea of testing at local pharmacies. This was viewed as an ideal location that would be accessible and comfortable, without stigma, but with the cleanliness and professionalism of a health care provider’s office.

### Preferred STI testing personnel

Many participants preferred to be tested by health care professionals, in keeping with the expressed preference for STI testing at private doctors’ offices. Although many participants noted that anyone can be trained to provide routine STI testing, they viewed health care professionals as having greater expertise to address a broad range of concerns.

*“I’ll have other questions when I go to get tested that I need them to answer*. *If it’s just somebody not trained, you can’t really answer all of my questions that I have.”*

The preference for testing by health care professionals may explain why testing at LGBT community centers was not valued more highly. Although participants reported appreciation for the role of these centers in their community, none expressed a preference for testing at them even though many reported having previously been tested there or having referred or accompanied other community members for testing at these locations.

For others, being respected and supported by the person conducting the test was more important than their formal training.

“I’m indifferent on who it is, I’m just here for the service, so as long as I’m respected and treated nicely and information’s confidential, we’re good.”

“I don’t want a health freak. (Moderator: What’s a health freak?)

Someone who’s all about health because they’ll look at you dirty. I feel like they look at you dirty like, Oh, you’re here so that means your not being healthy.”

### Preferred STI testing methods

When asked about the preferred STI testing method including urine, oral swab, urethral swab, rectal swab, and blood test, participants noted that urine sampling was the easiest though they believed that blood testing was most accurate. Few had undergone anal or penile swabs, but most said it was viewed as painful. Participants expressed confidence in the results of anal and penile swabs and that accuracy of results outweighs discomfort.

### Innovative ideas for routine STI screening

Incentives for testing, whether in the form of cash/gift cards or free condoms were strongly endorsed by all. Participants also felt that information on STI prevention, including details on how to obtain PrEP, should be offered in all testing settings to reinforce the critical message. Several responses reflected testing procedures that are more widely available in other areas, such as mobile testing units or reminders from the local Health Department to encourage routine testing. One participant mentioned STI testing kiosks [22], which he had seen in Europe, and other participants in his focus group strongly endorsed this idea. An STI testing kiosk provides clients with STI testing kits without any personnel involved, much like a vending machine. Several participants expressed an interest in having community health workers come to people’s homes to administer tests, which would preserve privacy while also ensuring the accuracy of results (major concern participants expressed regarding home testing). The most imaginative suggestion was for the development of a reusable at-home testing device, which the participant likened to a glucometer or Breathalyzer. Each of these suggestions further illustrated participants’ desire for diverse approaches to testing that emphasize privacy, control, and legitimacy.

## Discussion

Based on these findings, providing YB MSM in the Deep South with a variety of STI testing options that incorporate confidentiality, legitimacy, absence of stigma, and minimal barriers to access (e.g., insurance) will ensure a more patient-centered STI testing framework. This qualitative study of STI testing preferences of YB MSM in Birmingham demonstrates the importance of testing options to respond to the diverse needs of individuals with varying ages, preferences, and resources. Participants expressed a preference for traditional clinical encounters in private doctors’ offices, ideally conducted by a primary care physician (PCP) to ensure trust, confidentiality and legitimacy of the provider and testing process. A 2017 study in the UK also found that individual MSM had diverse needs and preferences with regards to STI-testing, and professionalism and expertise were valued qualities of clinic staff [23]. Alternative methods like self-testing and home testing, however, were not appealing to the study participants because concerns about the reliability of these novel methods outweighed privacy benefits. This contrasts with many studies in which self-testing in non-clinical settings was widely accepted or preferred by participants, including MSM, because of the ease and confidentiality of self-swabbing [24]. Finally, novel STI testing options were of interest to YB MSM including testing at retail pharmacies and even via STI kiosks [25], highlighting the demand for privacy, legitimacy, and client control. Because these strategies are novel, there is little data to support their acceptability from the perspective of young, black MSM.

The fact that a majority of participants preferred STI testing by a PCP is a new finding and underscores the need for access to healthcare and, specifically, PCPs for the sexual health of YB MSM. Another unexpected finding is that participants were less interested in STI self-testing, and, instead, wanted to be evaluated and tested by a physician. Although any physician was preferred, participants were especially interested in a PCP that they know and trust based on a longstanding relationship. Access to a PCP not only may allow more acceptable STI testing but may be protective against HIV for YB MSM. In one cohort of YB MSM in the Deep South, the lack of a PCP increased the odds of HIV seroconversion by six-fold [25]. It is likely that the benefits of a PCP are derived not just from the STI testing process but also from the consistent sexual health counseling and care by a trusted provider, which are essential to sexual health in this vulnerable group.

Faith-based organizations, local Health Departments, and other community partners often have the infrastructure and access to key populations, including YB MSM, needed to support STI testing initiatives, but study participants highlighted many challenges that must be addressed in order to leverage these community partners to fight against STIs in the Deep South. STI testing at churches was not acceptable to many who cited concerns about confidentiality and the willingness of churches to participate (due to stigma around STI/HIV). The greatest barrier to testing at the local Health Department was a perception of stigma, specifically around clinic staff and patient interactions. Participants reported stigma related to poverty, promiscuity, and homosexuality at the STI clinic at the local health department. Because many health departments provide affordable testing, it is critical to address these barriers, especially for YB clients, who are disproportionately affected by poverty and STIs [26].

Given the convenience and privacy that STI self-testing allows, it was surprising that participants were not enthusiastic about the option of collecting one’s own test at home or elsewhere. This finding is in contrast to multiple studies, which have demonstrated the acceptability and feasibility of STI self-testing in young persons, including university students and MSM at high risk for STIs [16, 17, 27]. This may stem from a lack of awareness and/or education about self-testing accuracy. Further, participants expressed concern that, following a positive STI self-test at home, some may not 1) seek the proper treatment, 2) change their sexual risk behavior, and/or 3) be emotionally prepared to deal with the diagnosis without the support of a medical provider. Before incorporating STI home testing into routine care in the Deep South, additional research is needed to explore concerns about inappropriate treatment and insufficient support for YB MSM with a positive STI test result at home.

This study has several limitations. We experienced difficulty recruiting YB MSM from gay bars, clubs, and community events focused on LGBT issues, thus the views expressed by our participants may not reflect the most hard to reach YB MSM. Yet, because of the age, demographic, and STI/HIV history of participants (Table 1), this sample consists of men at risk for STIs, including HIV, in the Birmingham, Alabama metropolitan area. This study includes many who are HIV positive and recruited from flyers at the UAB HIV/AIDS clinic, a clinical site which provides HIV treatment and prevention to high risk MSM (i.e. PrEP). For this reason, participants may be more oriented towards traditional medical clinics and less interested in pursuing STI testing at home. Notably, the UAB HIV/AIDS Clinic where many were recruited, serves a vulnerable (uninsured, impoverished) patient population including HIV positive and HIV negative clients eligible for PrEP and has high rates of STIs. Specifically, 30% of STI screening tests conducted on MSM receiving care in the UAB 1917 HIV/AIDS PrEP clinic are positive [28]. Of HIV-infected MSM receiving STI testing at this clinic, 17% had a positive test for *mycoplasma genitalium* [29]. Of all men with HIV receiving STI testing in this clinic, 9% had a positive test for gonorrhea, 7% had incident syphilis, and 3% had a positive test for chlamydia [30]. Recruiting from this population gives a voice to a high risk group of young men transmitting and acquiring STIs and HIV [31,32]. If we had been successful in our attempts to recruit participants from bars, adult bookstores, or community events, those individuals may feel less comfortable receiving STI testing in clinical sites and prefer testing at health fairs or venues that target young, gay men (e.g., bars, clubs). It is likely that participants’ recruitment methods (e.g., via flyers in clinics) also affected their views toward stigma and confidentiality. In other words, participants’ willingness to participate in a FG, openly discussing STI testing preferences, suggests that they may be less affected by stigma and more agreeable to STI testing, in general, than harder to reach YB MSM.

In closing, in order to increase STI testing among YB MSM in the Deep South we must employ testing strategies that are patient-centered, capitalizing on desires for privacy, legitimacy, and accessibility and alleviating barriers including stigma and excessive out of pocket costs. This will involve changes related to STI testing location (i.e., access, insurance), staff attitudes and behaviors (i.e. stigma), test collection, and counseling and support in the post-test period. There are also opportunities to expand existing services to include innovative testing at retail pharmacies and STI testing kiosks, which will require additional research and funding streams. Because the STI epidemic requires support from payers, state and federal governments (e.g., health departments), health systems and providers, halting STIs, including HIV, in YB MSM in the Deep South will take a concerted effort on the behalf of numerous stakeholders.

## Supporting information

S1 Fig Focus group script. (DOCX)

S2 Fig Focus group participant ages (years) according to session. (DOCX)
