# Supplementary material for: Do young black men who have sex with men in the deep south prefer traditional over alternative STI testing?
Source: PLoS One. 2018 Dec 27;13(12):e0209666. doi: 10.1371/journal.pone.0209666 (PMC6307723; doi:10.1371/journal.pone.0209666)
Supplement: S1 Fig — (DOCX) [file pone.0209666.s001.docx]

Supplemental File 1

Focus Group Script

Background:

Good Morning (Afternoon/Evening) and welcome to our session.

[Introduce self and co-moderator]

Thank you for volunteering to be a part of our study and taking the time to meet with us. The purpose of our study is to find out what STD screening services are most desirable for young men. STD means “sexually transmitted disease” and includes HIV, chlamydia, gonorrhea, syphilis, herpes and many other infections. Through this project, we hope to identify the STD screening services that young men would be most likely to use if or when they are concerned about an infection. The ultimate goal is to use your ideas and opinions to design sexual health services in a way that is most acceptable in order to increase our chances of diagnosing and treating these infections. By identifying and treating these infections, we can reduce the spread of STDs in young men which has been a challenge for many years. Today’s meeting will take about 75 minutes of your time (15 for consent and 60 for discussion).

You have been invited to attend because we feel your views on this topic are very important. You may choose to answer, or not to answer, any of the questions I ask. The session will be tape-recorded, and the written record of the tapes will provide helpful information that can be used to identify important goals and preferences related to sexual health. Names or other facts that could identify you will not be in the written record or in the reports based on this meeting. Also, the tapes will be erased and destroyed at the end of the study. You may see us taking some notes in case we have any problems listening to the tapes.

[Go over the the consent form; have the participants sign consents and distribute incentive money]

Focus groups are a little bit different from just sitting and talking and I’d like to tell you how:

1. There are no right or wrong answers to questions
2. When you disagree with something that is said, we really want to hear what you have to say, but it is important to be respectful in your disagreement.
3. There are no unimportant ideas
4. Be comfortable, feel free to get a drink or go to the restroom as needed

Because we are recording, it is very important for only one person to speak at a time. For reasons of confidentiality I will ask that you use only your participant number so we have some way of identifying the different responses. So, before you speak, please say your number like this “I am #5 and I think….”

Next, I would like to orient you to the types of STD services we will be discussing.

Types of STD testing. First, I would like to inform you of the types of STD samples on which testing can be performed. We can screen for some STDs in a person’s urine, others require a blood test, and some require swabs of body sites exposed during sexual intercourse. These swabs may be performed on the anus or rectum, inside the mouth or genital area.

Personnel collecting the test. These tests can be performed by medical provider or staff, non-medical staff and even patients themselves. Even the blood test can be done by a patient with a fingerstick test much like a person with diabetes pricks their finger to test their sugars. You may not be familiar with some of these newer tests such as fingerprick blood tests and swabs of the rectum because they are expensive and may not be routinely offered.

Location of testing. The patient can perform most of these tests at home. Alternatively, they can go to a medical clinic or health department for testing. Lastly, these tests can be offered at a community outreach program through a nonprofit organization. Some examples of community outreach would include STD testing at a health fair or public library.

1.When you think of the best possible place to test someone for STDS including HIV- where would it be?

PROBES:
A.Would you be more likely to go to a medical clinic or a non-medical site? Why?

B. If you had to choose medical clinic would you prefer a private clinic or a health department for STD testing? Why?

C. Would you be likely to go to a non-medical site like a health fair or a public library offering STD screening? Why?

D. Are there other types of medical or non-medical places that you would be likely to go for help if you thought you had an STD? Why?

E. If you could pick up a testing kit at a drug store, collect your own urine and rectal samples, and drop the samples off at a laboratory would you be likely to use that service? Why?

F. If you were able to perform all of these services on yourself, would you be likely to collect these tests? Why?

G. Would you like to collect them at home, in a medical clinic, or at a non-medical facility? Why?

H. Are there examples of places you would NOT go for testing?

2. When you think of the best possible way to test someone for STDS including HIV- who would be collecting the sample?

PROBES:

A. Would you prefer that a medical provider collect these specimens rather than a non-medical provider or staff person? Why?

B. Would you prefer that a non-medical person collect this sample or would you prefer to collect it yourself? Why?

C. Would you prefer to drop this sample off at a laboratory window or would you like to hand-deliver it to medical personnel? Why?

D. Would you prefer to deliver this sample to a medical personnel or to a staff person who has no medical background but is friendly and accepting? Why?

E. Would you like to receive the results via phone call or text message or would you like to hear your results in person from a medical provider? Why?

F. Are there examples of persons that you would NOT want to assist with testing?

3. When you think of the best possible way to test someone for STDS including HIV- how would it be collected (ie. urine, swab, blood test)?

PROBES:

1. If you had the choice between a swab of your rectum or a blood or urine test which would you choose? Why?
2. If you could design the perfect STD test what would it be? How would it be collected (ie. urine, blood, other) ? Who would collect it? Why?
3. Are there examples of tests that you would NOT want collected?
