# Supplementary material for: Do young black men who have sex with men in the deep south prefer traditional over alternative STI testing?
Source: PLoS One. 2018 Dec 27;13(12):e0209666. doi: 10.1371/journal.pone.0209666 (PMC6307723; doi:10.1371/journal.pone.0209666)
Supplement: S2 Fig — (DOCX) [file pone.0209666.s002.docx]

**Supplemental File 2.**

**Focus Group Participant Ages (years) according to session**

**Focus group 1 Focus Group 2**

**August 10, 2017 October 4, 2017**

32 23

26 23

27 29

29

28

31

**Focus Group 3 Focus Group 4**

**October 24, 2017 December 7, 2017**

21 24

33 21

30 27

18 22

26 30

25 19

19 34

18 25

35 27

24

**Focus Group 5**

**February 8, 2018**

17

32

24

23

33

22

23

35
